# Genomic Data: Building Blocks For Life Or Abstract Art?

**DOI:** 10.3389/frym.2024.1249534

**Published:** 2024-02-08

**Authors:** Rachel Horton, Kate Lyle, Susie Weller, Lisa Ballard, Anneke Lucassen

**Affiliations:** 1 Clinical Ethics, Law and Society (CELS), Centre for Human Genetics, Nuffield Department of Medicine, University of Oxford, Oxford, United Kingdom; 2 Centre for Personalised Medicine, Nuffield Department of Medicine, University of Oxford, Oxford, United Kingdom; 3 Clinical Ethics, Law and Society (CELS), Primary Care, Population Sciences and Medical Education, University of Southampton, Southampton, United Kingdom

## Abstract

The genes found in the genetic code (genome) are sometimes called the “building blocks for life” but knowing how they impact human health can be more complicated than it sounds. This article aims to show how difficult it can be to understand how our genes can affect our health, and why it is not always easy to work out a patient’s result from genetic tests. We follow the story of Ben, whose muscles have been getting weaker for a few years. To find out why, Ben has had his genetic code sequenced, and we will walk you through a process by which his results can be analyzed. Through this activity, we will show you that analyzing patients’ genome tests is a bit like interpreting abstract art, in which different people might see and value different things.

## DNA and Disease

The genes found in the genetic code, which is also called the genome, are sometimes referred to as the “building blocks for life”. But understanding how our genes impact our health can be more complicated than it sounds. Let us look at why.

Human DNA is 99.9% identical between people. In the 0.1% of DNA that is different between individuals, we can look for explanations for various diseases. But that 0.1% still contains 4–5 million differences in the DNA code, which are called variants—far too many to look through one by one. So, how do scientists and medical experts identify the DNA differences that impact human health?

Until recently, when a person was thought to have a genetic condition scientists and medical experts would guess which gene was most likely to be the cause, based on the person’s symptoms. They might then analyse that gene (if possible) and look for variants that might affect the way that gene worked. If they did not find a reason, they would pick another gene to examine. But since humans have thousands of genes, this was often a slow process!

Some genetic tests are still done in this way, but often, nowadays, people who are thought to have genetic conditions will have their entire genetic code analyzed—also known as whole genome sequencing [1]. This is a laboratory procedure in which data on the person’s entire genetic code are gathered and then explored, in the hope of revealing the cause of their health condition. An entire genetic code contains thousands of different genes and all the code in between the genes, too. Let us look at an example to see how the process works.

## Meet Ben

**Figure 1:**
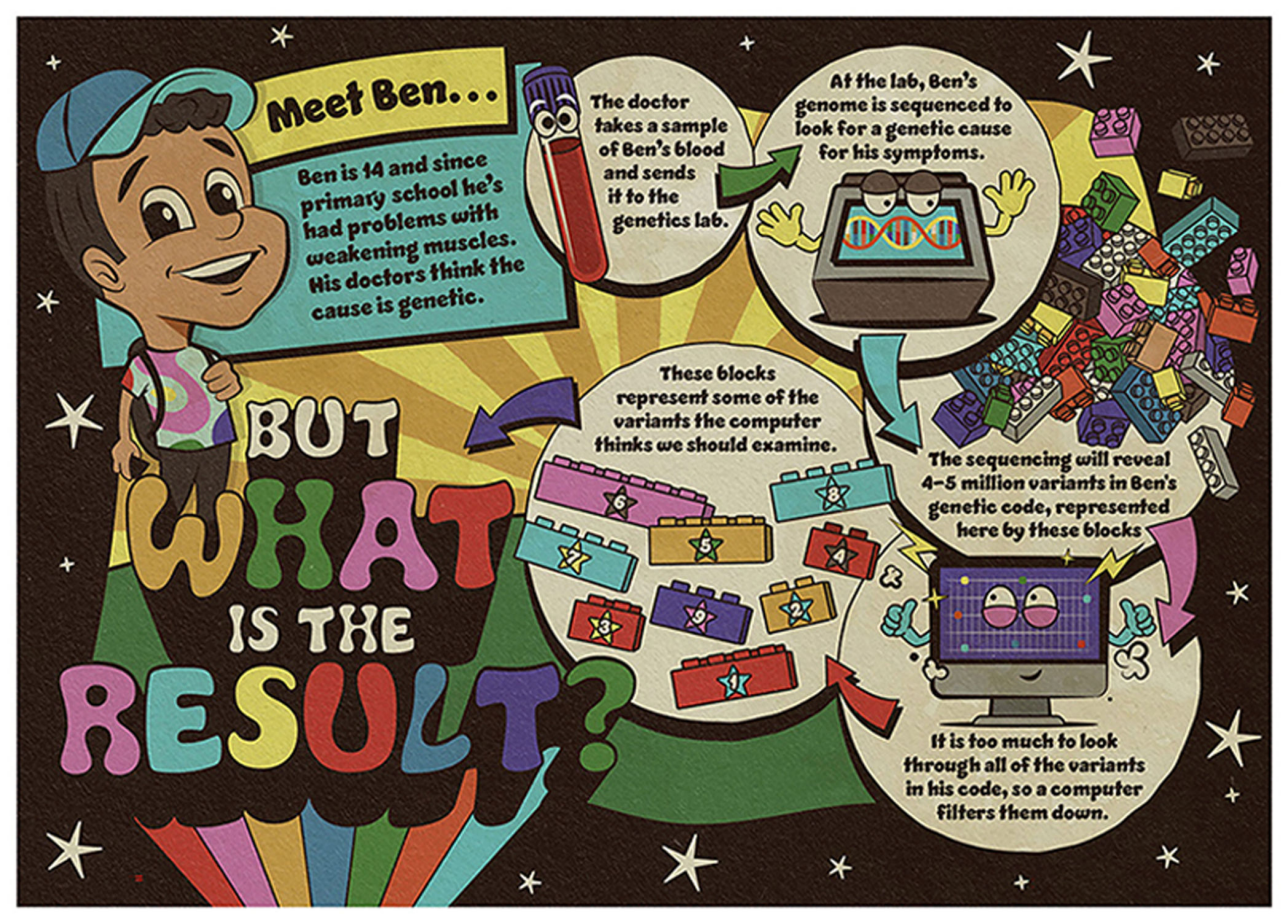
To try to find an explanation for his muscle weakness, Ben’s blood sample was sent to a genetics laboratory to sequence his genome. The sequencing revealed many variants in his genetic code. The variants identified by the computer as worthy of further examination are represented by the numbered blocks.

Ben is a young teenager whose muscles have been getting gradually weaker for a few years. He now has difficulty standing up and lifting things. Figure 1 describes the case of Ben, who has had his genome sequenced to look for a genetic explanation for this muscle issue.

The computer identified some variants of interest in Ben’s genetic code, but there are too many for them all to be likely to be a result. Sometimes genetic explanations are down to one variant, sometimes they are down to several variants in combination, and sometimes scientists and medical experts do not have enough data to rule out a genetic explanation. Somehow, they must decide the best way to filter the variants, to figure out what they might mean for Ben.

## Choosing What to Focus On

**Figure 2:**
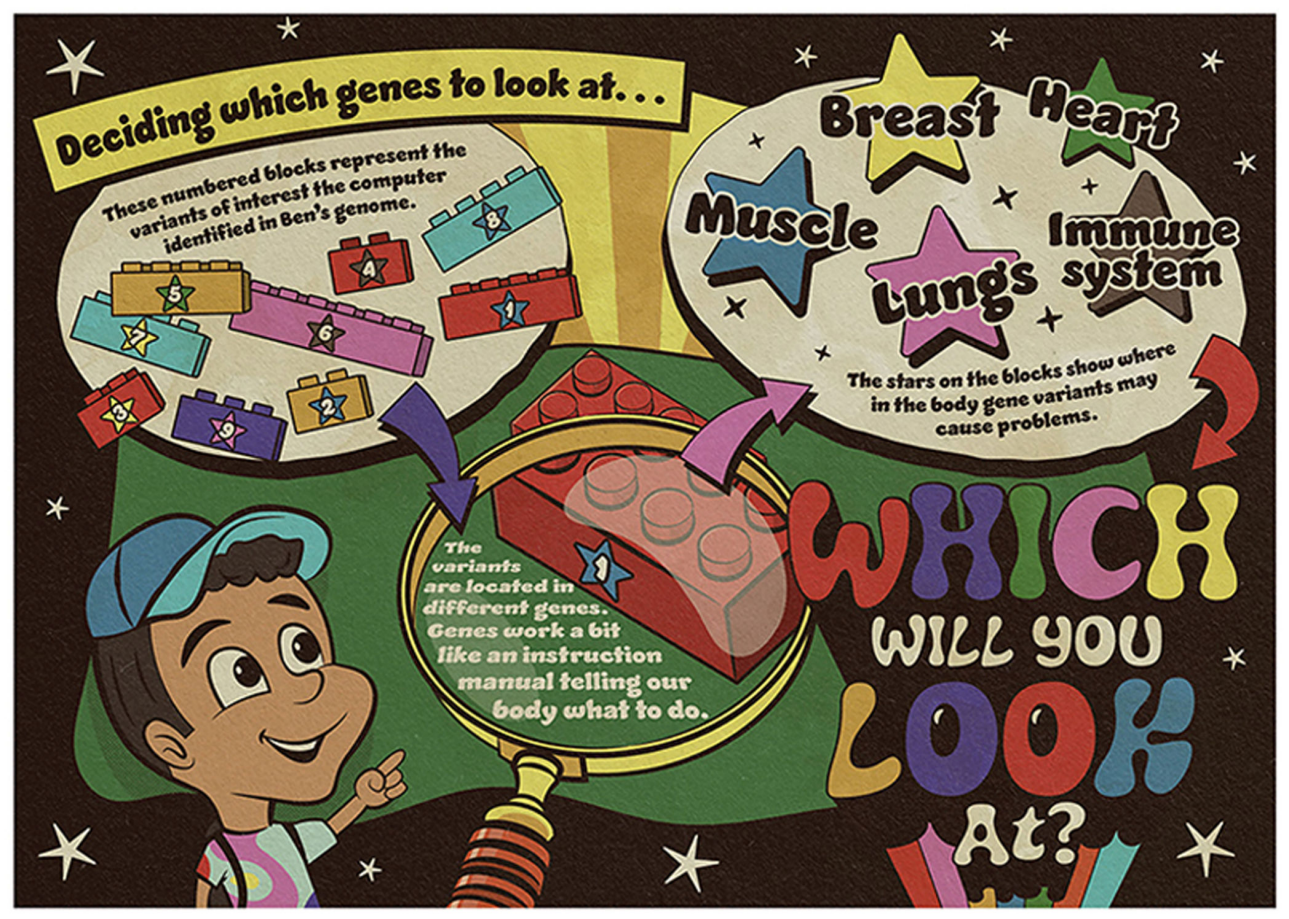
The numbered blocks represent the variants identified by the computer as worthy of further investigation. The variants are located in different genes, so scientists and medical experts must decide which genes to look at. The stars on each block indicate where in the body the gene variants may cause problems.

The variants found in Ben’s genome are located in many different genes. Some of the genes are *known* to be linked to muscle problems, and others *might* be, although it is not yet known how. One way to narrow down the number of variants to look at would be to look at only the genes known to be involved in the problem being studied. Figure 2 shows the genes that Ben’s variants are located in. Which do you think might be interesting to explore?

Since Ben visited his doctor because of weak muscles, scientists and medical experts might decide to look at the variants in genes that code for things related to muscles first. But other genes might still be important to study, as they regulate chemicals or enzymes that might affect muscles. For some genes it is not yet known what they do, or which parts of the body they work in.

**Figure 3:**
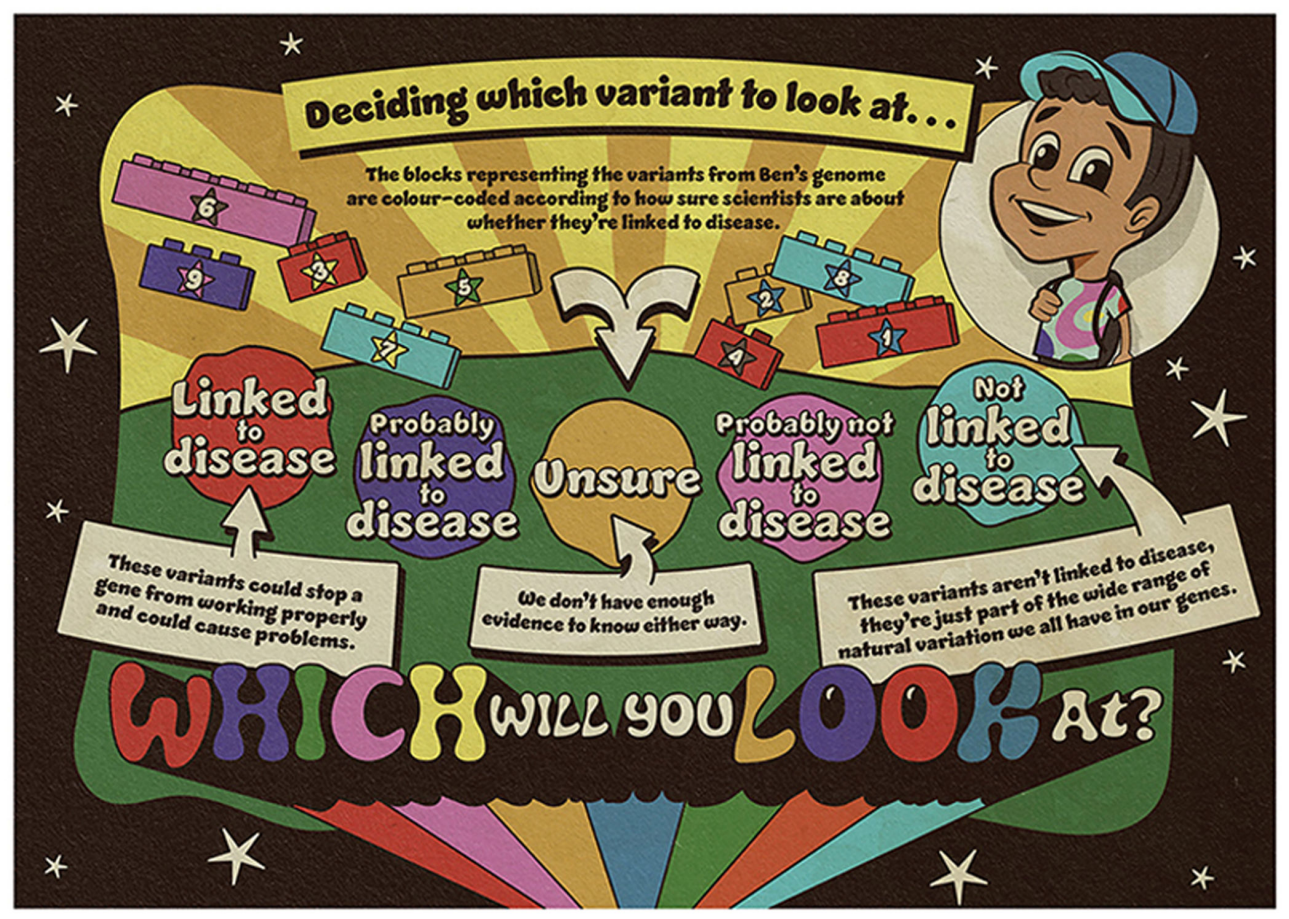
Scientists and medical experts need to decide which variants to look at. As before, the blocks represent the variants of interest identified through sequencing Ben’s genome. These are color coded according to how sure scientists and medical experts are about the strength of the evidence.

But now there is another decision to make. The scientific evidence we have about genetic variants, and how certain we are that they are linked to health conditions, varies. Figure 3 shows the different levels of evidence we have about the variants of interest from Ben’s genome.

As we learn more about the genetic code, the strength of the evidence might change (it can get stronger or weaker), but this might take many years. So, scientists and medical experts need to decide whether to look only at variants that are definitely linked to disease, or whether to broaden their search to study variants that they are less sure about. Which variants would you wish to look at? Would you only want to explore the ones that scientists and medical experts know are linked to disease, or would you also study some that they are less sure about?

## What Should We Tell Ben?

The variants that scientists and medical experts might choose to look at will depend on the patient’s specific circumstances. Let us look in more detail at some of the variants found in Ben’s genome and consider how relevant they might be to him. In the figures, each block represents a variant of interest identified in Ben’s genome. If we decide to look at variants in genes known to be related to muscle, and we have also chosen to exclude variants that are not known to be linked to disease, this would leave us with two types of variants:

- *Variant in a muscle gene, definitely linked to disease (block number 1)*.

This type of variant means Ben is a carrier for a condition that causes severe muscle weakness in babies. Being a carrier means that Ben does not have the condition and this variant is not the cause of his muscle weakness. For this variant to impact Ben’s health, he would have needed to inherit two copies—one from each parent—but Ben has only inherited one. If, in the future, Ben has a baby with someone who is also a carrier of this same muscle condition, there is a one in four chance their child will inherit both copies of the variant and, therefore, have the muscle condition that these variants are linked to. If Ben has a baby with someone who is not a carrier, their child will not be affected.

*Would you include this as part of Ben’s result?*

- *Variant in a muscle gene, but uncertain whether linked to disease (block number 2)*.

Some variants in this gene cause muscle weakness, but usually when this happens people have symptoms from birth. Ben developed symptoms when he was at primary school, suggesting his variant does not stop the gene from working in the same way.

*Would you include this as part of Ben’s result?*

## Looking Wider

We have looked in depth at a couple of Ben’s variants. Maybe choosing whether to report them to Ben was an easy choice for you, but maybe it was not. But what about the other variants that were filtered out along the way? Here are a few of them:

- *Variant in a breast gene, definitely linked to disease (block number 3)*.

*BRCA* variants are linked to cancer in adults, meaning that Ben will have a higher risk of developing breast cancer. But as he is male, the chances are much lower than if he were female. He will also have a higher chance of developing prostate cancer later in life, but cancer screening will not be available to him until he is at least 40 years old [2]. Is it important for Ben to know now that he might be at risk in the future, or might this cause him anxiety, especially when he cannot do anything about this risk?

Ben’s parents and siblings, and any future children Ben has, will each have a one in two chance of having the *BRCA* variant, too. Women with this sort of variant have a higher chance of developing breast and ovarian cancer as adults, and they would be offered extra screening and surgery to reduce this risk. But if this variant is not included as part of Ben’s result, his relatives would not know they had a high chance of having a *BRCA* variant predisposing them to cancer.

*Would you include this as part of Ben’s result?*

- *Variant in an immune system gene, definitely linked to disease (block number 4)*.

This variant means Ben could be seriously ill if he takes the medicine Abacavir, which is used for treating HIV. If Ben does not take the medicine, this variant will never make him ill. If Ben ever needed Abacavir, he would be tested for the variant first. The test is quick, so waiting for the results would not cause Ben any problems.

*Would you include this as part of Ben’s result?*

- *Variant in a heart gene, uncertain if linked to disease (block number 5)*.

This variant is in a gene for which other variants cause some people to develop thickening of the heart muscle, which can stop the heart from pumping properly. We do not know if Ben’s variant would do that. Ben could have an ultrasound scan to look at his heart, but this sort of heart problem can develop over time, so we still could not be sure that the variant would not cause future problems.

*Would you include this as part of Ben’s result?*

Including these variants as part of Ben’s result is not an easy decision. While these variants do not explain his muscle weakness, might this information be useful to him in other ways, and if so, when? The initial selection of which genes to focus on often helps scientists and medical experts avoid making these difficult decisions. These sorts of variants do not make it through the filtering process unless they are actively searched for. But at times, something like this slips through, and scientists and medical experts must decide whether the variant should form part of a person’s result or not.

## Constructing Ben’S Result

We worked through a similar example with visitors at a science festival in Southampton, UK. There were a range of views about what should be included in a patient’s result. Some people thought none of the variants should count as a result, while some wanted to include nearly all the variants they had studied. Sorting through the “building blocks” of life ended up feeling more like interpreting abstract art, with different people seeing and valuing different aspects.

You will also notice that, after all this work, we have not come up with a clear answer for Ben’s muscle weakness. Not getting a conclusive answer is the most common outcome for genome sequencing. As you have seen with Ben, there are different ways to look at genome data, and just because the entire code has been sequenced, it does not mean all the variants have been studied. Even for the variants that have been looked at, we do not yet know what they mean for Ben or his relatives [3, 4]. We hope that studying variants will find more answers…but perhaps it will raise more questions, too.

## Abbreviations

Genes: Sections of DNA that act as recipes for the proteins that make up our bodies. They influence characteristics (e.g., eye color) that we inherit from our (biological) parents.
Genetic code: The code in our DNA that provides instructions for the cells and organs of our body. We inherit these instructions from our parents.
GENOME: The entire genetic code of the DNA held in each cell.
DNA: The molecule inside cells that carries the genetic information that determines how our bodies grow and work.
Variant: A difference in the DNA sequence between people (like a different way of spelling a gene recipe). Only some variants will affect our health.
Genetic condition: A health condition caused (to a large extent) by variants in our genetic code (that may or may not be inherited from our biological parents).
Genome sequencing: A technique used to identify the exact order of the “letters” of the genetic code, or DNA. Variants in the genome might contribute to a health condition or disease.
Carrier: A person who has inherited one copy of a variant but the genetic condition in question requires a variant from each parent.
